# Supplementary material for: Remote care for mental health: qualitative study with service users, carers and staff during the COVID-19 pandemic
Source: BMJ Open. 2021 Apr 22;11(4):e049210. doi: 10.1136/bmjopen-2021-049210 (PMC8068948; doi:10.1136/bmjopen-2021-049210)
Supplement: Supplementary data [file bmjopen-2021-049210supp001.pdf]

Appendix 1

Interview topic guides for service users, carers, and staff

1 Service users

Mutual introductions.

Thank you for taking the time to speak with me today. Our conversation will be transcribed and anonymised and any identifiable data removed to protect your privacy.

You can stop the interview at any time if you would like a break or no longer wish to continue. If you were to become distressed or uncomfortable during the interview, what would be the best way for me to help? *(If appropriate, let interviewee know we can signpost to NHS or listening services)*

I will be available for a debrief after the interview if you feel you may benefit from it.

1. What motivated you to participate in this research? (*Warming up question – building rapport*)
2. *Researcher to ask participants how they are feeling about taking part in the interview – explore mood conversationally.*
3. Have you had to, or chosen to, self-isolate?
    - *If yes* – how has this been for you?
    - *If not* – are you able to go out and how have you found this?
4. Can you tell me about how mental health services usually support you?
    - What services do/did you access/ what treatment did/do you receive?
    - How often do/did you receive treatment?
    - When did treatment start (*and potentially end*)?

THIS.Institute

5. What access to care have you needed since the COVID-19 outbreak started?

6. [*If needed services could not be accessed*] Could you tell us what happened when you tried to access the service? What reasons were you given for the service being unavailable?

7. [*If needed services were accessed*] What changes in care have you experienced due to the COVID-19 outbreak, if any?

   Notes for researcher:
   - *Explore any change in usual treatment*
   - *Explore any change in frequency of treatment*
   - *Explore any interruption or delay of your usual treatments*
   - *Explore impact on the person*

8. How were these changes communicated or explained to you?

9. Were you offered a choice in how to communicate with services? If so what options were you given (for example via phone, video-calls email, text)?

10. If you now meet with you clinician online, how has this been for you? How are you finding these consultations?

    Notes for researcher:
    - *Explore how this change felt, what they did to support themselves through it (or others did)…*

11. Have you contacted support services that you would not normally use?
    - *Note for researchers: for example, NHS services (crisis support, 111, mental health out of hours support lines, home treatment team), non-statutory support (Samaritans, SANE, etc.), online support and forums.*

12. *If yes* – how have you found them? Do you think you will continue to use them?

13. Overall (*researcher to recap changes experienced*), how have these changes impacted you?

14. Is there anything you are finding particularly challenging?

15. Have any of the changes been positive for you?
    - Could they be maintained when the COVID-19 pandemic has passed?

16. Did you have any involvement in, or were you consulted on any changes in the provision of your care during the pandemic?
    - If yes – what worked well in the consultation

THIS.Institute

- If not – how might this have been done at the moment?

17. Who do you think should be prioritised for secondary mental health treatment during the COVID-19 pandemic?

    Notes for researcher:
    - *Explore why, what should guide decisions about prioritisation.*

18. Do you have any suggestions on how your care needs during the pandemic could be better met?

19. Is there anything that we haven't discussed that you think we should know in relation to this topic?

Ask interviewee how they feel after the conversation – explore mood conversationally.

Reminder of payment modality – a member of the research team will contact you via email.

THIS.Institute

2 Carers

Mutual introductions.

Thank you for taking the time to speak with me today. Our conversation will be transcribed and anonymised and any identifiable data removed to protect your privacy.

You can stop the interview at any time if you would like a break or no longer wish to continue. If you were to become distressed or uncomfortable during the interview, what would be the best way for me to help? *(If appropriate, let interviewee know we can signpost to NHS or listening services)*

I will be available for a debrief after the interview if you feel you may benefit from it.

1. What motivated you to participate in this research? (*Warming up question – building rapport*)

2. *Researcher to ask participants how they are feeling about taking part in the interview – explore mood conversationally.*

3. Can you tell me about your usual role as a carer?

   - Do you live with [*the person they care for - person name*]?
   - What mental health difficulties does [*person name*] experience?
   - What secondary mental health services do they usually access/ what treatment do they receive?
   - When did they start (and potentially stop) accessing services?

4. Have you and [*person name*] had to, or chosen to, self-isolate?

   - *If yes* – how has this been for you?
   - *If not* – are you able to go out and how are you both finding this?

5. Has your role as a carer changed during the COVID-19 pandemic, and if so how? How did you experience this change?

6. What access to care has the person you care for needed since the COVID-19 outbreak started?

7. [*If needed services could not be accessed*] Could you tell us what happened when you and the person you care for tried to access the service? What reasons were you given for the service being unavailable?

8. [*If needed services were accessed*] What changes in the care offered to [*person they care for]* have you noticed during the COVID-19 pandemic? How have they affected you? How have they been experienced by the person you care for?

THIS.Institute

Notes for researcher:

- *Explore any change in usual treatment*
- *Explore any interruption or delay of your usual treatments*
- *Explore impact on the carer*

9. How were these changes communicated or explained to you and *[person name]*?

10. Has [*person name*] received online consultations and, if so, how has this impacted your caring role? What do you think about online consultations?

    Notes for researcher:

    - *Explore how this change has felt, what carer did to support this, what adjustments were needed*

11. Overall (*researcher to recap changes experienced*), how have these changes impacted you?

12. Have you had to make any adjustments and, if so, how have these affected you?

13. Is there anything you are finding particularly challenging?

14. Have any of the changes been positive for you and/or the person you care for?
    - Could they be maintained after the COVID-19 pandemic has passed?

15. Did you have any involvement in, or were you consulted, on any changes to the provision of mental healthcare during the pandemic?
    - If yes – what worked well in the consultation
    - If not – how might this have been done at the moment?

16. Who do you think should be prioritised for secondary mental health treatment during the COVID-19 pandemic?

    Notes for researcher:

    - *Explore why, what should guide decisions about prioritisation.*

17. Do you have any suggestions on how your needs as a carer during the pandemic could be better met?

18. Is there anything that we haven't discussed that you think we should know in relation to this topic?

Ask interviewee how they feel after the conversation – explore mood conversationally.

.

THIS.Institute

3 Members of staff

Mutual introductions.

Thank you for taking the time to speak with me today. Our conversation will be transcribed and anonymised and any identifiable data removed to protect your privacy.

You can stop the interview at any time if you would like a break or no longer wish to continue. If you were to become distressed or uncomfortable during the interview, what would be the best way for me to help? *(If appropriate, let interviewee know we can signpost to NHS or listening services)*

I will be available for a debrief after the interview if you feel you may benefit from it.

1. Can you give me a quick background to your role? What kind of things would you typically do on a day-to-day basis?

2. How has the COVID-19 pandemic affected your role? *(Including whether they have been working from home)*

3. What pressures are you experiencing as a result of the COVID-19 pandemic?

4. How has the pandemic affected referrals into your service, in particular for service users that were known to the service before?
    - Have you noticed any changes about *how* people are coming into contact (via A&E, crisis teams, etc.)?

5. *[For hospital staff]* Have decisions about hospital admissions or discharge of service users been affected by the COVID-19 pandemic, in your opinion? How so?
    - Who makes these decisions?
    - How are these decisions affecting you?

6. How is COVID-19 affecting decisions on which service users to prioritise?
    - How are these decisions affecting you?
    - Do you feel that any particular patient groups may be particularly compromised?

7. How are decisions about what type of treatment to offer (including a shift to online consultations) being affected by the COVID-19 pandemic?

8. If some of your work has shifted to online consultations, how are you finding these?

9. How do you support mental health patients displaying COVID-19 symptoms?
    - How is it the decision made to refer them to acute healthcare settings?

THIS.Institute

10. Have the processes for referral to specialist services (e.g. occupational therapy) changed? How so?

11. Considering the changes we have discussed, are you facing any ethical or moral dilemmas which you were not confronted with before? *(Explore how these are experienced)*

12. How is this new way of working affecting you and your wellbeing? How is it affecting your life outside work (e.g. family arrangements, lifestyle, etc.)?

13. Has there been any changes to the support you are able to access in relation to your wellbeing and ability to provide support to others?

    - Has the COVID-19 pandemic affected your access to supervision and reflective practice, and if so how?
    - Are there processes in place to report or ask for support about the concerns you may be having?

14. How do you feel that service users are finding to the new ways of receiving care?

15. Has any chance in the service introduced due to COVID-19 had a positive impact?
    - Would these changes be maintained in the aftermath of the acute phase of the pandemic?

16. Is there anything that I haven't asked you that you feel we should know in relation to this topic?
